# A case of antisynthetase syndrome

**DOI:** 10.1002/ccr3.2778

**Published:** 2020-06-03

**Authors:** Clio P. Mavragani, Haralampos M. Moutsopoulos

**Affiliations:** ^1^ Departments of Physiology and Pathophysiology School of Medicine National and Kapodistrian University of Athens Athens Greece; ^2^ Chair Medical Sciences/Immunology Athens Academy Athens Greece

**Keywords:** antibodies against cellular antigens, antisynthetase syndrome, interstitial lung disease, Raynaud's

## Abstract

Respiratory complaints alone or in association with musculoskeletal complaints can be the predominant presenting feature of antisynthetase syndrome. Therefore, antibodies to cellular antigens should be evaluated in such clinical settings.

## Graphical abstract

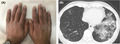

A 54‐year‐old man presented with a recent onset of arthralgias in both wrists and knees. System review revealed Raynaud's phenomenon and dry cough started approximately 3 months ago. Physical examination was significant for left knee arthritis, swollen hands, flat violaceous papules mainly on the dorsum of the metacarpophalangeal joints (Figure 1A), crackles over the left lower lung field, and 4/5 motor strength in upper extremities. Laboratory testing disclosed mild leucopenia with CK and aldolase levels being slightly elevated. High‐resolution computed tomography disclosed ground glass opacities, resulting in obscuration of the vascular outlines and adjacent airway walls, most likely consistent with organizing pneumonia (OP) (Figure 1B). Bronchoalveolar lavage revealed a lymphocytic exudate with cultures for common bacteria, fungi, and mycobacteria reported negative. Patient refused lung biopsy. Immunological testing was significant for positive ANA (titer 1/1280, cytoplasmic pattern), autoantibodies against Ro52 and Jo‐1 t‐RNA synthetase. The patient was started on methylprednisolone 0.4 mg/kg body weight and mycophenolate mofetil at a dose of 2 gr daily. This case highlights the significance of evaluating serum autoantibodies in patients presenting with subtle autoimmune manifestations not fulfilling classification criteria of an autoimmune disease.^1^ Moreover, the unusual presentation of antisynthetase syndrome as OP‐like picture is emphasized.^2^

**Figure 1:**
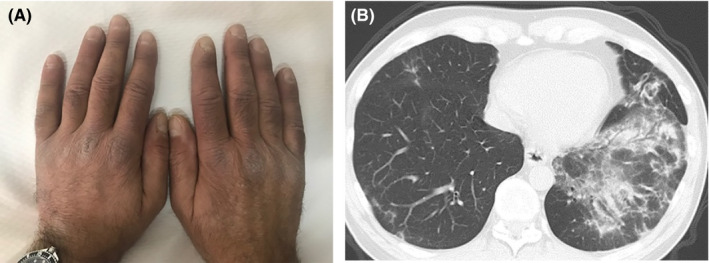
A, Flat violaceous papules on the dorsum of the metacarpophalangeal joints. B, High‐resolution computed tomography disclosed ground glass opacities likely consistent with organizing pneumonia

## CONFLICT OF INTEREST

None declared.

## AUTHOR CONTRIBUTIONS

CPM: wrote the initial draft, participated in reviewing the literature, interpretation of clinical findings, critical revision of the manuscript for important intellectual content, and approval of the final version. HMM: wrote the initial draft, participated in collecting patient data (pictures and clinical history), reviewing the literature, interpretation of clinical findings, critical revision of the manuscript for important intellectual content, and approval of the final version.
